# Prevalence of potentially inappropriate medications according to STOPP-Frail criteria in nursing home residents, the SHELTER study

**DOI:** 10.1186/s12877-024-05450-y

**Published:** 2024-10-26

**Authors:** Alireza Malek Makan, Hein van Hout, Graziano Onder, Harriet Finne-Soveri, Daniela Fialova, Rob van Marum

**Affiliations:** 1 Amsterdam University Medical Center, Departments of General Practice and Medicine for Older Persons, Vrije Universiteit, Amsterdam Public Health Research Institute, Meibergdreef 9, Amsterdam, The Netherlands; 2 Flevoburen: Geriatric Rehabilitation Centre, Vivium Zorggroep, Almere, The Netherlands; 3 Department of Geriatrics, Neuroscience, and Orthopedics, Agostino Gemelli University Hospital, Università Cattolica del Sacro Cuore, Rome, Italy; 4 Department of Wellbeing, National Institute for Health and Wellbeing, Helsinki, Finland; 5 Department of Social and Clinical Pharmacy, Faculty of Pharmacy, Department of Geriatrics and Gerontology, 1st Faculty of Medicine, Charles University, Staré Město, Czech Republic; 6 Department of Geriatric Medicine, Jeroen Bosch Hospital, s-Hertogenbosch, The Netherlands

**Keywords:** Potentially inappropriate medications, Nursing home residents, Adverse effects, End-of-life, Cognitive impairment

## Abstract

**Objective:**

The aim of this study was to determine the prevalence of potentially inappropriate medications (PIMs) in nursing home residents across eight countries and investigate differences between residents with and without cognitive impairment, as well as those with and without life expectancy of six months or less.

**Methods and deign:**

The study utilized the second edition of the STOPP-Frail criteria to operationalize PIMs in the baseline assessment of nursing home residents participating in the Services and Health for Elderly in Long TERm care (SHELTER) project. The data were collected between 2009 and 2012. The project was conducted in eight countries: Czech Republic, England, Finland, France, Germany, Italy, the Netherlands, and Israel. Cognitive impairment was measured by the cognitive performance scale (CPS). The presence of end-stage disease with a life expectancy of six months or less was recorded. The study included residents aged 60 years or older who underwent a valid medication assessment.

**Results:**

Among the 3,832 eligible residents, 87.9% had at least one PIM. Specifically, 24.3%, 23.5%, 18.8%, and 19.3% of residents had one, two, three, and four or more PIMs, respectively. On average, each person was prescribed 2.16 PIMs. Cognitively impaired residents (*n* = 1999) had an average of 1.96 PIMs (SD 1.49) per person, while residents with a low CPS score (*n* = 1783) had an average of 2.40 PIMs (SD 1.57) per person, showing a statistically significant difference (*P* < 0.001). Similarly, NH residents with life expectancy of six months or less had an average of 1.66 PIMs (SD 1.30), whereas those without had an average of 2.17 PIMs (SD 1.55) (*p* < 0.001). The average number of PIMs varied across countries, ranging from 3.23 in Finland to 2.15 in the UK (*P* < 0.001). Anti-platelets and aspirin were the most prescribed PIMs, accounting for over 38.0% of prescriptions.

**Conclusions:**

This study highlights the high prevalence of PIMs among nursing home residents. However, PIMs were somewhat lower in residents with cognitive impairment and life expectancy of six months or less. Efforts must continue to improve the rationale behind prescribing practices in nursing homes.

## Introduction

Ageing is often accompanied by an increased prevalence of chronic diseases, leading to a higher utilization of medications [1]. A significant portion of these medications are prescribed with the intention of mitigating the risk of future adverse health events. However, as individuals approach the end of life, the appropriateness of using preventive medications becomes a subject of debate, as the potential long-term benefits may no longer outweigh the associated adverse effects [2, 3]. This may be especially true for patients with a very limited life expectancy or end stage neurodegenerative disease. Given the high probability of medication becoming inappropriate in these situations, we hypothesized that cognitive impairment and limited life expectancy should be associated with a lower prevalence of PIMs.

Among older adults residing in nursing homes, especially those with progressive disorders like dementia, life expectancy is typically limited. Across six European countries, the proportion of deaths occurring within one year of admission to long-term care facilities ranged from 32 to 63%. The average length of stay in these facilities ranged from 6 months to 2 years [4]. Polypharmacy and hyper-polypharmacy (the use of ten or more medications) are prevalent among older nursing home residents [5, 6]. Studies have reported persistently high utilization of potentially inappropriate medications, including in nursing home patients with dementia, up until a few weeks prior to death [7]. 

Explicit screening tools such as the STOPP-START and Beers criteria have been developed to aid prescribers in identifying potential inappropriate medication use in older individuals. In 2017, the STOPP-Frail criteria were introduced as an explicit screening tool specifically designed to recognize inappropriate medication use in patients with a limited life expectancy [8]. An updated version of the STOPP-Frail criteria was published in 2020 [9, 10]. These criteria were developed to assist physicians in discontinuing potentially inappropriate medications in older individuals with end-stage irreversible conditions, poor one-year survival prognosis, severe functional impairment, severe cognitive impairment, or a combination thereof, with a focus on symptom control rather than disease prevention [8–10]. 

The objective of this study was to determine the prevalence of potentially inappropriate medications (PIMs) among nursing home residents across eight countries and investigate the differences between residents with and without cognitive impairment, as well as those with and without end-stage diseases. We hypothesized that cognitive impairment and limited life expectancy would be associated with a lower prevalence of PIMs.

## Methods and materials

### Study setting and residents

The data for this study was obtained from the Services and Health for Elderly in Long TERm care (SHELTER) project, conducted between 2009 and 2012. The project aimed to validate the interRAI instrument for long-term care facilities (interRAI-LTCF) [11]. The interRAI-LTCF is a standardized assessment tool used to evaluate the care needs and preferences of nursing home (NH) residents, aiding in care planning. It encompasses over 250 items that capture comprehensive information on residents, including sociodemographic variables, clinical characteristics (such as physical and cognitive status, clinical diagnoses), current service utilization, and medication use. The study included 4,156 residents from 57 NHs across 8 countries (Czech Republic, England, Finland, France, Germany, Italy, the Netherlands, and Israel). NHs that routinely utilized the interRAI-LTCF were selected as study partners in each country, without random selection, aiming to represent diverse NHs.

#### Eligibility

Both existing residents and those *entering the home* during the 3-month enrolment period within the homes were assessed using the interRAI-LTCF. Reassessments were conducted at 6 and 12 months for residents still in the facility. For this study, the baseline assessment of each participant was utilized. The study included residents aged 60 years or older with a valid medication assessment, without any specific exclusion criteria. A valid medication assessment comprised both the medication name and the ATC code.

#### Ethical approval

To conduct the study was given by the Ethics and Research Committee of Catholic University of the Sacred Heart of Rome, Italy. Ethical approval was obtained also from the Ethics Committees of the participating centres (Univerzita Karlova, Czech Republic; Universitaet Ulm, Germany; Terveyden Ja Hyvinvoinnin Laitos, Finland; Assistance Publique Hopitaux De Paris, France; University of Haifa, Israel; University of Kent, United Kingdom; Vereniging Voor Christelijk Hoger Onderwijs Wetenschappelijk Onderzoek En Patientenzorg, The Netherlands) and written informed consent was obtained from all participants. All procedures performed in this study involving human participants were in accordance with the ethical approval and standards of the local ethics committees. Regarding informed consent, both opt in and opt out procedures were followed.

### Determinants

#### Medication use

Was recorded in the interRAI-LTCF, considering the medications taken by each resident in the three days preceding each assessment. Medication data was gathered and verified from multiple sources, including physician order sheets and medication administration records. The information collected included proprietary names, Anatomical Therapeutic and Chemical (ATC) codes provided by the World Health Organization Collaborating Centre for Drug Statistics Methodology, formulation, dosage, frequency, and route of administration. Additionally, information was collected regarding medications taken on an “as-needed” basis.

#### Cognitive impairment

Was assessed using the Cognitive Performance Scale (CPS), which evaluates decision-making capacity, memory impairment, level of consciousness, and executive function. The CPS score ranges from 0 (indicating intact cognition) to 6 (representing severe cognitive impairment) [12]. The CPS has been validated in various studies [12, 13,]. The CPS has comparability with the Mini Mental State Examination (MMSE) a score ≥ 2 being associated to a diagnosis of dementia or a MMSE score of ≤ 19 [14]. So the CPS is used quite widely and the cut-off value of 2 or more is associated with cognitive impairment and dementia and therefore we considered 2 as the cut-off and cognitive impairment was considered present if the CPS score exceeded 2, indicating moderate or more severe cognitive impairment.

#### Life expectancy

Life expectancy of six months or less according to best clinical judgment and supported by a diagnosis of end-stage disease and communicated with the patient or their family [15].

### Outcome

#### Potentially inappropriate medications (PIMs) according to the 2nd edition of the STOP frail criteria

The selection and compilation of PIMs were based on the explicit criteria for PIM use in frail older adults with limited life expectancy, known as the STOPP-Frail-2 list, specifically sections B to J [9, 10]. For each section, the medications were converted to Anatomical Therapeutic and Chemical (ATC) codes, utilizing levels 3 to 5 depending on the specific criterion, in accordance with the codes provided by the World Health Organization Collaborating Centre for Drug Statistics Methodology. The ATC codes are all included and listed in Table 1. It should be noted that we did not have access to information regarding the indications for medication use or specific clinical conditions, which prevented the operationalization of section A. Section A defines PIMs in general terms, encompassing medications that residents persistently fail to take or tolerate without a clear indication or for symptoms that have already resolved (refer to Table 1). However, all SHELTER residents fulfilled the general pre-conditions, namely (i) requiring assistance with activities of daily living, (ii) having severe irreversible frailty, and (iii) being under the care of a physician who would not be surprised if the individuals died within the next 12 months.

**Table 1:** PIM prevalence according to stop-frail version2 in all residents, residents with vs. without cognitive impairment, with longer versus shorter life expectancy*

		All	CPS			Life expectancy in months		
	PIM (ATC code)	(*n* = 3832)	> 2 (*n* = 1999)	0–2 (*n* = 1783)	*P* value	< 6 months (*n* = 120)	> 6 months (*n* = 3698)	*P* value
Section B: Cardiac system	Lipid- lowering therapies (C10AA, C10AB)	10.3 (396)	7.2 (143)	14.0 (250)	< 0.01	5.8 (7)	10.5 (388)	ns
	Antihypertensive therapies (C03A, C03BA, C03EA, C03B, C08CA, C08GA01, C08GA02, C09BB, C09BX01, C09XA53, C09)	35.7 (1369)	28.3 (566)	44.7 (797)	< 0.01	20.8 (25)	36.3 (1341)	< 0.01
	Anti-anginal therapy (nitrates, nicorandil, ranolazine), (C01DA, C01DX16, C01EB18)	8.1 (312)	6.4 (127)	10.2 (181)	< 0.01	3.3 (4)	8.2 (305)	ns
Section C: Coagulation system	Anti-platelets (B01AC) And Aspirin (N02BA01, A01AD05)	38.5 (1475)	35.3 (706)	42.0(757)	< 0.01	20.8 (25)	39.0 (1444)	< 0.01
Section D: Central nervous system	Neuroleptic- antipsychotics (N05A)	20.6 (790)	26.6 (532)	14.0 (250)	< 0.01	20.8 (25)	20.6 (763)	ns
	Memantine (N06DX01)	4.4 (170)	6.1 (121)	2.7 (49)	< 0.01	0.8 (1)	4.5 (167)	ns
Section E: Gastrointestinal system	Proton pump inhibitors (A02BC)	35.7 (1368)	31.7 (634)	40.4 (720)	< 0.01	42.5 (51)	35.4 (1308)	ns
	H2 receptor antagonist (A02BA)	1.7 (64)	1.2 (2.4)	2.1 (38)	< 0.05	3.3 (4)	1.6 (59)	ns
Section F: Respiratory system	Theophylline (R03D)	2.2 (84)	1.8 (36)	2.5 (45)	ns	5.0 (6)	2.1 (78)	ns
	Leukotriene antagonist (R03DC)	0.3 (10)	0.2 (4)	0.3 (6)	ns	0.0 (0)	0.3 (10)	
Section G: Musculoskeletal system	Calcium_supplements (A12AX)	8.5 (324)	8.2 (163)	9.0 (161)	ns	2.5 (3)	8.6 (309)	< 0.05
	Vitamin D (ergocalifero and calciferol) (A11CC01, A11CC, M05BB03, M05BB04, M05BB05, M05BB06, M05BB07, M05BB08, M05BX53)	8.1 (309)	7.6 (152)	8.7 (157)	ns	3.3 (4)	8.1 (299)	ns
	Anti-resorptive/bone anabolic drugs for osteoporosis (bisphosphonates, strontium, teriparatide, denosumab), (M05B)	4.5 (172)	3.1 (62)	6.1 (109)	< 0.01	3.3 (4)	4.5 (168)	ns
	Long-term oral nonsteroidal anti-inflammatory drugs (M01A)	3.6 (138)	2.1 (42)	5.4 (96)	< 0.01	4.2 (5)	3.6 (133)	ns
	Long-term oral corticosteroids (H02)	3.5 (134)	3.0 (59)	4.2 (74)	< 0.05	7.5 (9)	3.4 (125)	ns
Section H: Urogenital system	Drugs for benign prostatic hyperplasia (5-alpha reductase inhibitors (G04CA, G04CB)and alpha-blockers (C02CA, C02LE)	3.3 (126)	2.9 (58)	3.8 (68)	ns	2.5 (3)	3.3 (123)	ns
	Drugs for overactive bladder (muscarinic antagonists and mirabegron (A04AD01, G04BD12)	0.1 (4)	0.1 (2)	0.1 (2)	ns	0.0 (0)	0.1 (4)	ns
Section I: Endocrine system	Anti-diabetic drugs (A10AD, A10B)	10.7 (411)	9.3 (185)	12.4 (221)	< 0.01	10.8 (13)	10.7 (397)	ns
Section J: Miscellaneous	Multivitamin combination supplements (A11)	10.6 (407)	9.9 (198)	11.6 (207)	ns	3.3 (4)	10.7 (396)	< 0.01
	Folic acid (B03BB, B03AD, B03AE01, B03AE02, L01BA, V04CX02)	5.8 (221)	5.9 (118)	5.5 (98)	ns	5.8 (7)	5.8 (214)	ns

Ns = not significant (*P* > 0.05)

CPS = Cognitive Performance Scale

*Life expectancy less than 6 months with documented end stage disease and discussed with resident and/or relatives

### Data analysis

The data analysis was conducted using Statistical Package for the Social Sciences (SPSS) version 26 (SPSS Inc, Chicago, IL, USA). Prevalence was reported as frequency and percentage. Continuous variables were presented as mean with standard deviation (SD). Differences in prevalence were assessed using Chi-square tests for categorical variables and T-tests for continuous variables. Statistical significance was set at a p-value below 0.05.

## Results

**Fig. 1:**
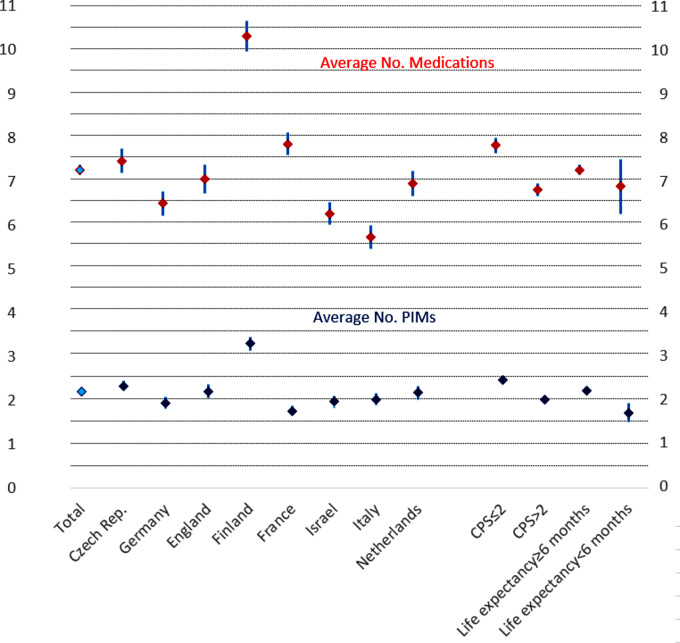
Average number of PIMS (upper panel) and number of drugs (lower panel) across countries with 95% Confidence Intervals, level of cognitive impairment (CPS) and life expectancy*. CPS = Cognitive Performance Scale, > 2 reflects moderate to severe impairment. *Life expectancy less than 6 months with documented end stage disease and discussed with resident and/or relatives

Of the 4156 residents included in the SHELTER study, 324 did not meet the eligibility criteria; hence, 3832 residents were included in this study. Of 324 residents not included, 96 were under 60 years, and another 228 had no valid medication registration. The average age of the residents was 84 years, ranging from 60 to 110 years, with 74% being women. On average, residents were taking 7.2 medications (SD 3.5) (Table 1; Fig. 1). Between 2.2% and 4% of the medications were prescribed as needed. Residents with moderate to severe cognitive impairment (*N* = 1999, 52.2%) used fewer medications and PIM`s compared to those without. Medication use of residents with a life expectancy of less than six months was not statistically different from those with a longer life expectancy (Table 2). In addition, 80% of the residents with a life expectancy of less than six months, used one or more PIM versus 86% in those with a longer life expectancy (p=0.06).

**Table 2:** Mean number of all medication vs. PIM`s in all residents, residents with vs. without cognitive impairment, residents with lower and longer life expectancy*

	Total Sample (*N* = 3832)	Cognitive impairment Moderate-severe (CPS > 2, *n* = 1999) No-mild (CPS ≤ 2, *n* = 1783)	Life expectancy* < 6 month (*n* = 120) ≥ 6 month (*n* = 3698)
Mean no of drugs used (SD)	7.20 (3.50)	6.75 (3.33) 7.77 (3.61) *P* < 0.001	6.81 (3.49) 7.20 (3.50) Ns
Mean no of PIMs (SD)	2.16 (1.54)	1.97 (1.50) 2.4 (1.57) *P* < 0.001	1.66 (1.31) 2.17 (1.55) *P* < 0.001

CPS = Cognitive Performance Scale

*Life expectancy less than 6 months with documented end stage disease and discussed with resident and/or relatives

On average, residents had 2.16 PIMs (SD 1.54). Among the residents, 87.9% had at least one PIM, with 24.3%, 23.5%, 18.8%, and 19.3% having one, two, three, or four or more PIMs, respectively. The percentage of residents with one or more PIMs was 83.1% in those with cognitive impairment (*n* = 1999) compared to 89.4% in those with no or mild cognitive impairment (*n* = 1783) (*p* < 0.0001). Additionally, residents with cognitive impairment had a lower average number of PIMs: 1.96 (SD 1.49) compared to 2.40 (SD 1.57) in residents without cognitive impairment.

Similarly, residents with the shortest life expectancy (< 6 months) had a lower average number of PIMs compared to those with a longer life expectancy  1.66 (SD 1.30) versus 2.17 (SD 1.55) (Table 2).

Regarding the types of PIMs, anti-platelet drugs and antihypertensive therapies (AHT) were the most common PIMs with preventative indications, accounting for 38.5% and 35.7% of PIMs, respectively. Although these drugs were less frequently prescribed to cognitively impaired residents (CPS > 2) and residents with a shorter life expectancy, their prevalence still exceeded 20% in both groups. Proton pump inhibitors and antipsychotic drugs, commonly used for symptomatic purposes, were also highly prevalent at 35.7% and 20.6%, respectively. Antipsychotic drugs, however, had a higher prevalence in patients with cognitive impairment (26.6% versus 14.0%, *p* < 0.05) (Table 2; Fig. 1).

**Fig. 2:**
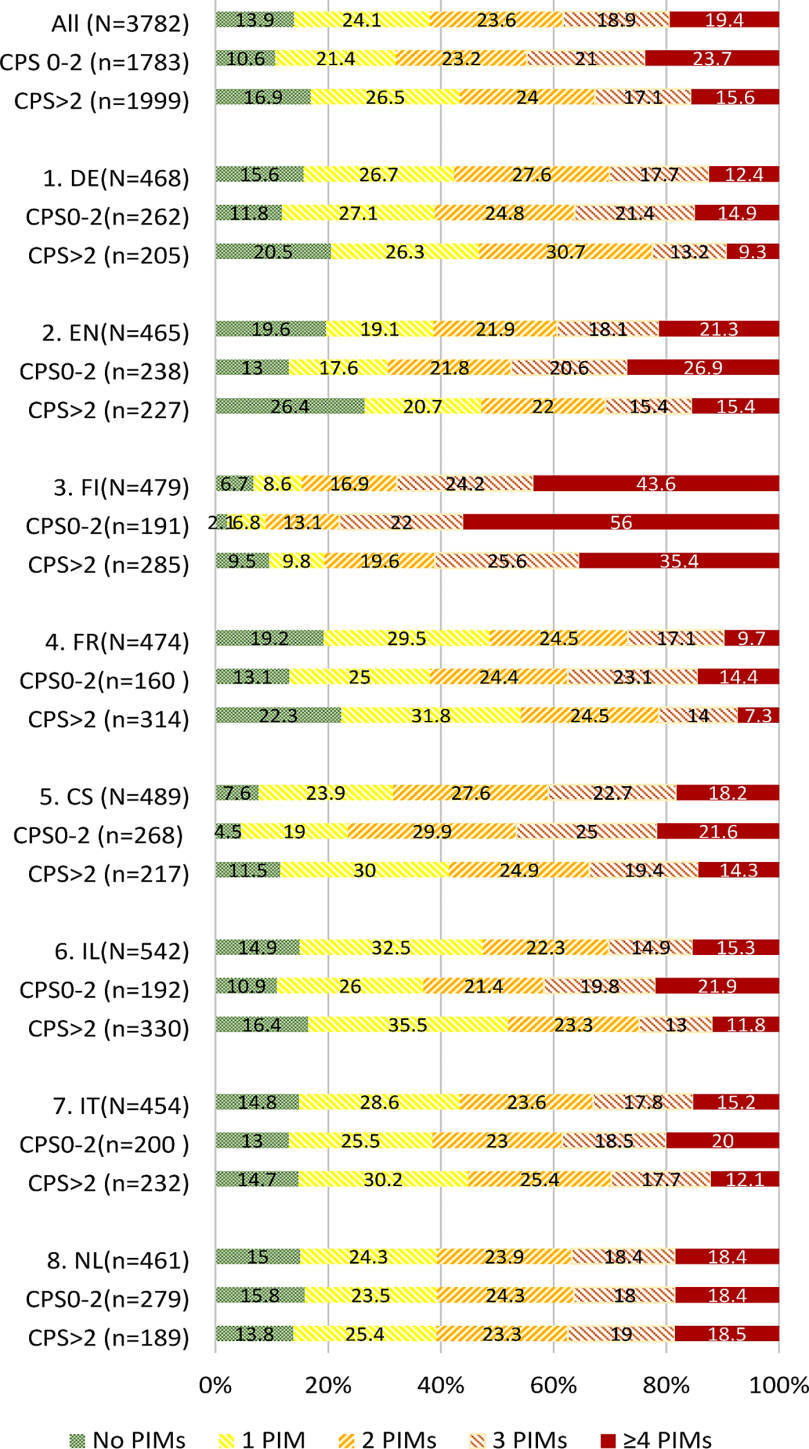
PIM prevalence in residents with and without cognitive impairment across countries. CPS = Cognitive Performance Scale > 2 indicates moderate to severe impairment. CS = Czech Republic, DE = Germany, EN = England, Fin = Finland, FR = France, IL = Israel, IT = Italy, NL = the Netherlands. * *P* < 0.0001 (comparison between high and low CPS on percentage of PIMs)

The prevalence of residents with one or more PIMs varied across countries, ranging from 93.3% in Finland to 80.4% in England (Figures. 1 and 2). In six countries, the group of residents with cognitive impairment had a larger proportion without PIMs compared to those without cognitive impairment (*P* < 0.001). Only in Italy and the Netherlands, no significant difference was found. There were no cross-country differences in PIM prevalence between residents with longer and shorter life expectancies. Due to the low percentage of residents with limited life expectancy, between country comparison of PIM prevalence was not feasible for this item.

In Finland, residents with cognitive impairment (CPS > 2) had significantly higher use of the following medications compared to other countries (*P* < 0.01): anti-anginal therapy (27.0% compared to less than 5.0% in most other countries), calcium supplements (26.3% vs. less than 10% in other countries), calciferol (38% vs. less than 10% in other countries), and multivitamin supplements (43.2% vs. less than 10% in all other countries). These medications were also more frequently prescribed to residents with lower CPS scores compared to other countries. Osteoporosis medication use in Finland differed substantially from use in all other countries. Almost two thirds of the residents used at least one of these medications. This was mainly the result of a high vitamin D use (65.6%), but also calcium use was higher than in other countries (29%). Interestingly, in Finland, the use of these medications was significantly higher among residents with a life expectancy of over 6 months compared to the same group in other countries.

## Discussion

This study presents data on the prevalence of inappropriate medications in European nursing home residents, based on the 2nd edition STOPP-Frail criteria. The results reveal that PIMs (Potentially Inappropriate Medications), as defined by the STOPP-Frail criteria, are present in nearly 90% of the residents within a typical European sample of nursing home residents. It is noteworthy that, on average, two out of seven prescribed medications were considered PIMs. As expected, our study found a lower prevalence of PIMs in patients with cognitive impairment compared to those with no or mild cognitive impairment, as well as in residents with a life expectancy of less than 6 months. This indicates that physicians appear to exercise more caution when prescribing medications for individuals with advanced dementia or poor prognosis. It is possible that because of the role physicians have in both (de)prescribing and estimating life expectancy, this strengthened this association. There seems to be an association between the average number of PIMs and the overall number of medications prescribed. However, it is concerning that 15.4% of nursing home residents with cognitive impairment still had four or more PIMs. Moreover, over 20% of the residents were using antihypertensive medications or platelet aggregation inhibitors, and over 10% were using lipid-lowering medication. Even in residents with a life expectancy of less than six months, the average number of PIMs remained high at 1.66. These findings align with previous studies highlighting the continued use of medications in the last years or months of life [16, 17]. 

While this study provides valuable insights, it is important to acknowledge its limitations.

Although the Shelter study data collection took place between 2009 and 2012, the data is still relevant. More recent studies on PIM prevalence based on STOPPFrail use in nursing homes are in accordance with our results. Recent data shows a prevalence of PIM use between 85% and 96% [18–21] in nursing home residents which is close to what we found (around 87%). Since other studies only look at data in 1 country at the time, we feel that the data in our study comparing data from 8 countries and many residents adds knowledge to existing data.

Based on the ADL scale, not every resident in the Shelter study appears to meet the STOPFrail criteria (as opposed to STOPP criteria) [22]. At the same time, it is expected that admission to a nursing home is evidence of vulnerability that will increase over time and lead to death. A recent study showed that almost 65% of patients awaiting long-term care are eligible for the application of STOPPFrail criteria with over 90% prescribed at least one PIM. We expect that in case of people already admitted to long-term care the percentage of patients meeting STOPPFrail criteria will be larger than the mentioned 65%. However, we realize that using these criteria on all residents in this study may overestimate the number of patients with inappropriate medication.

The low percentage of residents with a limited life expectancy in this study seems surprising but can be explained by the way this item is defined in the interRAI. While many studies only require the doctor’s best clinical judgement to determine life expectancy, the interRAI also requires that 2 other conditions are met: it must be supported by a diagnosis of end-stage disease and communicated with the patient or their family. We believe that these last 2 criteria often are not met. For instance, an earlier SHELTER study showed that 86.4% of residents close to death did not receive an accurate prognosis [23]. Detailed clinical information such as blood pressure or the specific indications for drug prescriptions was not available, which may also have resulted in an overestimation of the actual number of PIMs. Additionally changes in medication use over time were not studied for individual patients during their stay in nursing homes, potentially missing any decreases in prevalence among cognitively affected residents. Due to the cross-sectional nature of our study, we could not say anything about the course of medication use, although given the including data after 3 months of admission to the nursing home, it can be assumed that the first medication review had already taken place.

We were not able to operationalize all STOPPFrail criteria specifically. Therefore, the number of PIMS may be overestimated.

Finally, the convenience sampling method used for participant selection limits the generalizability of the conclusions to the participating countries.

*Strengths* of this study include the use of a multinational cohort and the application of the internationally well-validated interRAI standard for monitoring. This ensured reliable and fair comparisons [11]. Moreover, the large sample size enabled meaningful comparisons between residents with and without PIMs.

What are the implications of this study? First, these findings raise questions regarding medication prescriptions for nursing home residents, particularly those in the end-stage of life or with end-stage dementia, where the potential health benefits over a median life expectancy of 1 to 2 years need to be carefully weighed against the risks of adverse drug events. While deprescribing appears to be feasible and relatively safe towards the end of life [24], this study suggests that it is not a common practice. Clinicians often find it challenging to discontinue medications, particularly those related to coagulation. Various factors contribute to this phenomenon, as suggested in the literature [25, 26]. Some physicians believe there is sufficient evidence to justify medication prescriptions, while others are hesitant to change medications initiated by their colleagues. Lack of education and experience in safely deprescribing, difficulty in communicating deprescribing decisions, and concerns about “giving up on the patient” and hastening death are other barriers. Additionally, clinicians may feel pressured to adhere to clinical guidelines that often lack evidence from studies involving older populations with limited life expectancies. For instance, the use of psychotropic drugs remains common in home-dwelling individuals with mild dementia, despite limited evidence of their benefit [27]. Patients and their relatives may also express anxiety about discontinuing medications they have been using for many years [28]. 

For nursing home residents, prescribing physicians must carefully consider the expected treatment benefits while considering the short life expectancy and the risk of side effects [29]. 

## Conclusion

This study conducted as part of the SHELTER study suggests a high prevalence of PIMs among nursing home residents across countries. While PIMs were somewhat lower in residents with cognitive impairment and those with end-stage diseases, there is an ongoing need for efforts to improve rational prescribing in nursing home settings and reduce potential adverse effects.

## Data Availability

Data are not publically accessible due to privacy regulations. Reasonable requests for data access and material can be directed to the corresponding author.
